# Glutamine/glutamate metabolism rewiring in reprogrammed human hepatocyte-like cells

**DOI:** 10.1038/s41598-019-54357-x

**Published:** 2019-11-29

**Authors:** Maria Ballester, Enrique Sentandreu, Giovanna Luongo, Ramon Santamaria, Miguel Bolonio, Maria Isabel Alcoriza-Balaguer, Martina Palomino-Schätzlein, Antonio Pineda-Lucena, Jose Castell, Agustin Lahoz, Roque Bort

**Affiliations:** 1 Unidad de Hepatología Experimental, CIBERehd, Instituto de Investigación Sanitaria La Fe, Hospital Universitari i Politècnic La Fe, 46026 Valencia, Spain; 2 Unidad de Descubrimiento de Fármacos, Instituto de Investigación Sanitaria La Fe, Hospital Universitari i Politècnic La Fe, 46026 Valencia, Spain; 3 Unidad de Biomarcadores y Medicina de Precisión and Unidad Analítica, Instituto de Investigación Sanitaria La Fe, Hospital Universitari i Politècnic La Fe, 46026 Valencia, Spain

**Keywords:** Regenerative medicine, Metabolomics, Reprogramming

## Abstract

Human dermal fibroblasts can be reprogrammed into hepatocyte-like (HEP-L) cells by the expression of a set of transcription factors. Yet, the metabolic rewiring suffered by reprogrammed fibroblasts remains largely unknown. Here we report, using stable isotope-resolved metabolic analysis in combination with metabolomic-lipidomic approaches that HEP-L cells mirrors glutamine/glutamate metabolism in primary cultured human hepatocytes that is very different from parental human fibroblasts. HEP-L cells diverge glutamine from multiple metabolic pathways into deamidation and glutamate secretion, just like periportal hepatocytes do. Exceptionally, glutamine contribution to lipogenic acetyl-CoA through reductive carboxylation is increased in HEP-L cells, recapitulating that of primary cultured human hepatocytes. These changes can be explained by transcriptomic rearrangements of genes involved in glutamine/glutamate metabolism. Although metabolic changes in HEP-L cells are in line with reprogramming towards the hepatocyte lineage, our conclusions are limited by the fact that HEP-L cells generated do not display a complete mature phenotype. Nevertheless, our findings are the first to characterize metabolic adaptation in HEP-L cells that could ultimately be targeted to improve fibroblasts direct reprogramming to HEP-L cells.

## Introduction

Human fibroblasts have been directly converted into hepatocyte-like cells (HEP-L) cells by the expression of a combination of transcription factors^1–4^. *In vitro* characterization of HEP-L cells usually include hepatic-specific functional assays such as synthesis and secretion of albumin and α1-antitrypsin, glycogen storage, indocyanine green and LDL transport and/or phase I and phase II metabolic activities, yet the metabolic dynamics to support these new cellular demands remain unexplored.

Proliferating human fibroblasts display a high metabolic rate due to the massive metabolic requirement to replicate their whole cellular components. Contact-inhibited, quiescent dermal fibroblasts, maintain high metabolic rates explained by continuously degrade and resynthesize their macromolecules and cellular components, as well as enhanced biosynthesis of extracellular matrix components^5^. In fact, dermal fibroblasts divert glutamine/glutamate to proline biosynthesis, a crucial aminoacid in collagen synthesis (28% of proline and hydroxyproline)^6^.

Liver plays a critical role in the homeostasis of various nutrients in the body, thus representing the main site of control of inter-organ intermediate metabolism^7^. Hepatocytes are responsible for handling ammonia generated in peripheral tissues. Highly toxic free ammonia is transported to the liver as glutamine. Once in the liver, periportal hepatocytes deamidate ammonia by a liver-specific glutaminase (GLS2), not inhibited by glutamate concentration^8,9^, releasing ammonia and glutamate, the latter being partially secreted. Glutamate generated by these upstream periportal hepatocytes is in part captured by perivenous hepatocytes coupled to glutamine synthesis and release, supporting an interorgan glutamine flux^10,11^. Another important destination of glutamate in hepatocytes is citrate through reductive carboxylation of α-ketoglutarate to generate lipogenic acetyl-CoA in the cytoplasm^12^.

Here we demonstrate using untargeted and targeted stable isotope-labeling metabolomics, that glutamine/glutamate metabolism in HEP-L cells reflects that of hepatocytes and away from the parental cell type. Still, we detect lower rate of reductive carboxylation attributed to an incomplete metabolic rewiring of reprogrammed HEP-L cells.

## Results

### Untargeted metabolite profiling of 24-hour cultured cell media

**Figure 1:**
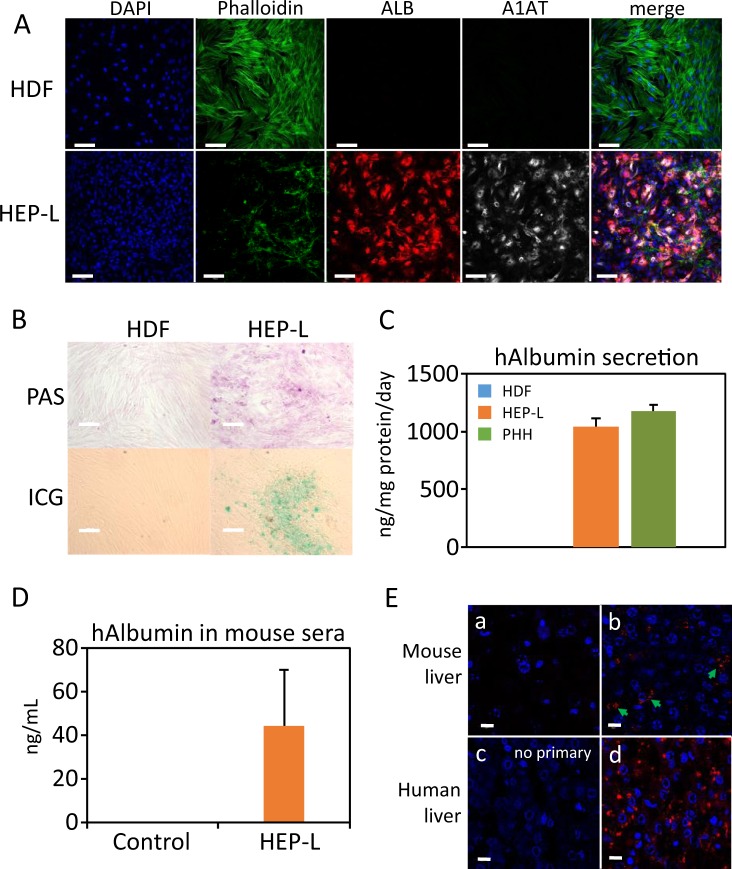
HEP-L cells activate the hepatic program and perform basic hepatic functions. (**A)** Representative fluorescence images of HDF and HEP-L cells immunostained with antibodies against albumin and α1-antitrypsin. Phalloidin-488 was used to visualize F-actin. Nuclei were stained with DAPI. Bar equals 100 µm. **(B)** PAS staining and indocyanine green of HDF and HEP-L cells. **(C)** Human albumin contained in 24-hour incubated cell media was quantified by ELISA. Primary cultured human hepatocytes were used as control. **(D)** Human albumin present in sera from HEP-L cells transplanted SCID mice was quantified by ELISA (n = 5). **(E)** Human-specific albumin immunofluorescence staining of liver formalin-fixed sections. Panel a: non-transplanted mice; panel b: HEP-L cells transplanted mice; panel c: control human liver (no primary antibody); panel d: human liver. Notice that the pattern of expression of human albumin in engrafted HEP-L cells is very similar to human hepatocytes in liver i.e. punctuated dots scattered throughout the cytoplasm.

Human dermal fibroblasts (HDF) were reprogrammed to hepatocyte-like cells (HEP-L) cells as described elsewhere^2^. HEP-L cells expressed hepatic gene programs (Supplementary Fig. S1) and displayed functions characteristic of hepatocytes such as expression of albumin, α1-antitrypsin, glycogen storage and indocyanine green transport (Fig. 1A–C) as well as low expression of α-fetoprotein (Supplementary Fig. S1B). When transplanted into mice with paracetamol-induced acute liver failure, mice sera contained human albumin and cells were able to colonize the liver (Fig. 1D–E).

**Figure 2:**
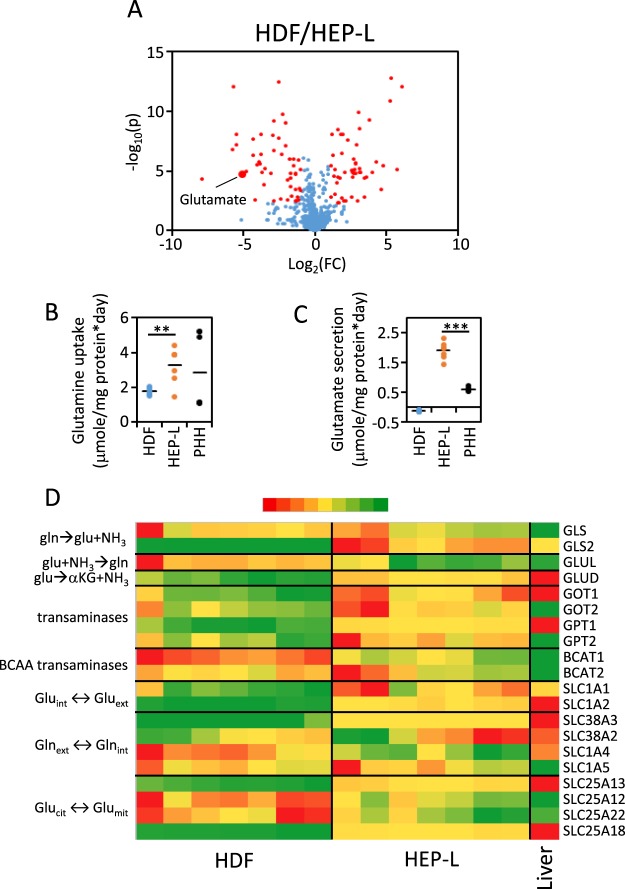
Untargeted metabolomics analysis of 24-hour culture cell supernatant. (**A)** Volcano plot of metabolites/features identified in cell media recovered after 24 hours. Data was analysed using batch Molecular Feature Extraction (MFE) included in Profinder B.08.00 Software (Agilent). Plotted metabolites/features were present in all samples of at least one group (HDF or HEP-L cells) and the cutoff MFE score was 95 (max 100). Glutamate identity was validated with a chemical standard and mass spectra. N = 7 per group. **(B)** Glutamine uptake was determined by subtracting glutamine concentration in media from control plates (without cells) and 24-hour incubation media. Values are individually plotted. N = 7 per group. **(C)** Glutamate secretion was determined by subtracting glutamate concentration in 24-hour incubation media and media from control plates (without cells). Values are individually plotted. N = 7 per group. **(D)** Heatmap representation of mRNA levels related to glutamate/glutamine metabolism and transport determined by qRT-PCR in HDF and HEP-L cells and plotted as relative gene expression (Liver = 1). Numerical data provided in Table S6. N = 7. **p < 0.005; ***p < 0.001.

To obtain a broad picture of the metabolic changes induced after reprogramming to HEP-L cells, we performed an untargeted metabolite profiling of cell media incubated for 24 hours with HDF or HEP-L cells. A list of 858 features/ metabolites (characterized by exact mass and retention time) were selected (see methods for details). Peak intensity values were analyzed in a volcano plot (Fig. 2A). One hundred and one metabolites were differentially concentrated in cell media (Table S1; FC > 2; FDR < 0.005). A preliminary identification of these metabolites was performed using HMDB database (https://www.hmdb.ca) with an accuracy of 10ppm. The identity of glutamic acid (exact mass: 147.0531) was unequivocally confirmed using a chemical standard.

Differential glutamate concentration in cell media was validated in a new set of experiments (data not shown). In parallel, we estimated the rates of glutamine and glutamate uptake/secretion by HDF and HEP-L cells normalizing per mg of protein since HEP-L cells are 3.8-fold bigger and contain 4.1-fold more protein per cell (Supplementary Fig. S2). Glutamine consumption was 2-fold higher in HEP-L cells reaching a value comparable to primary cultured human hepatocytes (PHH) (Fig. 2B). In parallel, glutamate utilization changed dramatically. While HDF were net consumers of glutamate from cell media (HMM media contains glutamate), HEP-L cells, as well as PHH, were net producers (Fig. 2C). These changes run in parallel to profound transcriptomic remodeling of genes involved in glutamine/glutamate metabolism and transport, such as those involved in deamidation (GLS2), glutamine synthetase (GLUL) and genes involved in glutamate to α-ketoglutarate conversion i.e. glutamate dehydrogenase (GLUD) and liver-specific transaminases (GOT and GPT) (Fig. 2D).

### Glutamate flux reversal in HEP-L cells

**Figure 3:**
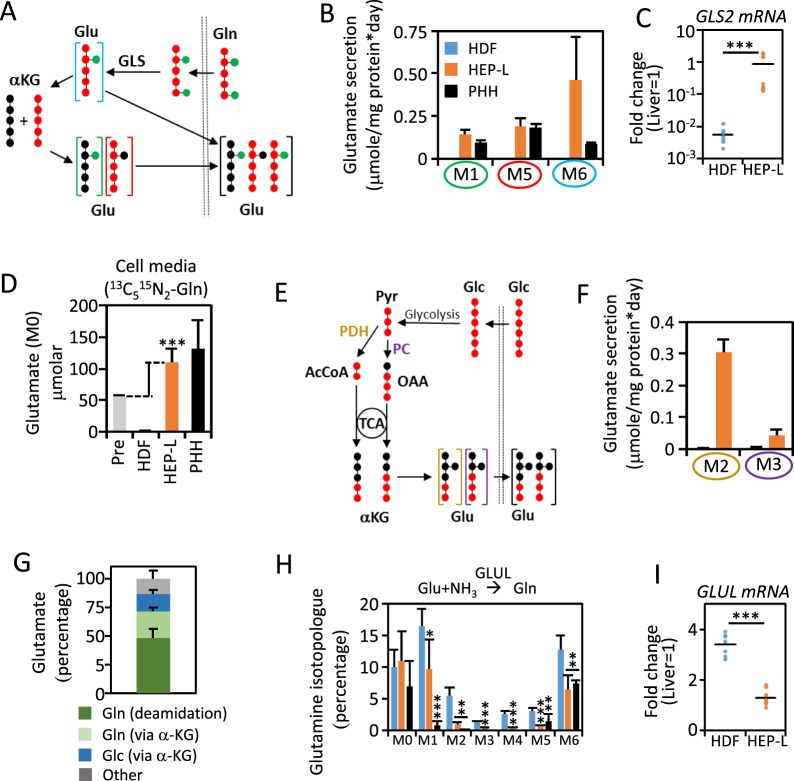
HEP-L cells secrete glutamate derived from multiple sources. (**A**) ^13^C_5_^15^N_2_-Gln is uptaken and deamidated to ^13^C_5_^15^N-Glu (blue circle), that can be released back to the media or deaminated to ^13^C_5_-αKG. ^13^C_5_-Glu (red circle) is produced by transamination of the latter with unlabeled amino. ^15^N-Glu (green circle) is produced by transamination of unlabeled αKG with glutamine-derived ^15^N. **(B)** Cells were incubated with HMM media containing 2 mM ^13^C_5_^15^N_2_-Gln and labeled glutamate quantified by LC-MS in the supernatant after 24 hours. Exact mass of glutamate (M1) corresponds to ^15^N-Glu (Table S2). **(C)** Levels of *GLS2* mRNA were determined by qRT-PCR in HDF, HEP-L cells and individually plotted as relative to human liver gene expression. N = 7. Liver corresponds to pooled mRNA from 5 different donors. **(D)** Cells were incubated with HMM media containing 2 mM ^13^C_5_^15^N_2_-Gln. Glutamate (M0) was quantified in the supernatant after 24 hours by LC-MS. HMM cell media contains 54 μM unlabeled glutamate (M0). **(E)** ^13^C_6_-Glc is uptaken and metabolized to ^13^C_3_-Pyr which is incorporated into the TCA cycle via pyruvate dehydrogenase (PDH) or pyruvate carboxylase (PC) to ultimately generate ^13^C_2_-Glu (golden circle) or ^13^C_3_-Glu (purple circle) respectively. **(F)** Cells were incubated with HMM media containing 3.15 g/L ^13^C_6_-Glc and the concentration of labeled glutamate quantified by LC-MS in the supernatant after 24 hours. **(G)** Carbon moiety source of secreted glutamate in HEP-L cells. **(H)** Intracellular isotopologue distribution of glutamine isotopologues (M1-M6) as a readout of glutamate to glutamine conversion in HDF, HEP-L cells and PHH incubated with 2 mM ^13^C_5_^15^N_2_-Gln. **(I)** Levels of *GLUL* mRNA were determined by qRT-PCR in HDF, HEP-L cells and individually plotted as relative to human liver gene expression. N = 7. Liver corresponds to pooled mRNA from 5 different donors. Data in panels B, D, F and H is represented as mean ± s.d. from a total of 15 replicates per group (HDF and HEP-L cells) and 7 replicates for two experiments/donors (PHH). *p < 0.05; **p < 0.005; ***p < 0.001.

Net production of glutamate in cell media from HEP-L cells can only be explained by de novo synthesis and secretion. Glutamine is the main cellular source of glutamate. The fate of the carbon and nitrogen moiety of glutamine present in the media was established by incubation with [U-13C, 15 N] glutamine (^13^C_5_^15^N_2_-Gln). ^13^C_5_^15^N_2_-Gln is transported into cells, where it is deamidated to ^13^C_5_^15^N_1_-glutamate (^13^C_5_^15^N-Glu; M6) by glutaminases (Fig. 3A). Non-proteinogenic glutamate is largely converted to α-ketoglutarate via either glutamate dehydrogenase or transaminases. We found accumulation of ^13^C_5_^15^N-Glu, ^13^C_5_-Glu (M5; originated from transamination of ^13^C_5_-α-ketoglutarate (α-KG) with unlabeled nitrogen) and ^15^N-Glu (M1; originated from transamination of unlabeled α-KG with glutamine-derived ^15^N) in the cell media from HEP-L cells and primary cultured human hepatocytes, but not from HDF (Fig. 3B; Table S2). Accumulation of glutamate in cell media was corroborated by ^13^C-NMR of replicate experiments (Supplementary Fig. S3). As expected, such difference cannot be attributed to a lack of glutaminase activity in HDF (Supplementary Fig. S4), although, de novo expression of phosphate-activated glutaminase (GLS2) at levels comparable to PHH could explain this change (Fig. 3C).

Interestingly, unlabeled glutamate present in ^13^C_5_^15^N_2_-Gln tracing media was consumed by HDF, but secreted by HEP-L cells (Fig. 3D), indicating the novo generation of glutamate from non-glutamine sources. Glutamate can also be synthesized from glucose through glycolysis coupled to the TCA cycle (Fig. 3E). We found a significant accumulation of ^13^C_2_-Glu and ^13^C_3_-Glu in cell media from HEP-L cells, but not from HDF incubated with ^13^C_6_-glucose (^13^C_6_-Glc; Fig. 3F). By combining the data collected from incubations with ^13^C_5_^15^N_2_-Gln and ^13^C_6_-Glc, we estimated that de novo synthesized and secreted glutamate originates from glutamine (71%) and glucose (15%). Contribution from non-glutamine non-glucose sources may account for another 15% approximately (Fig. 3G). We observed a low capacity to produce glutamine by glutamate-ammonia ligation catalyzed by glutamine synthetase (GLUL) in HEP-L cells (Fig. 3H). A metabolic feature shared with PHH. In parallel, *GLUL* mRNA levels in HEP-L cells were reduced 3-fold, similar to PHH levels (Fig. 3I).

### Glutamine utilization is reduced in HEP-L cells

**Figure 4:**
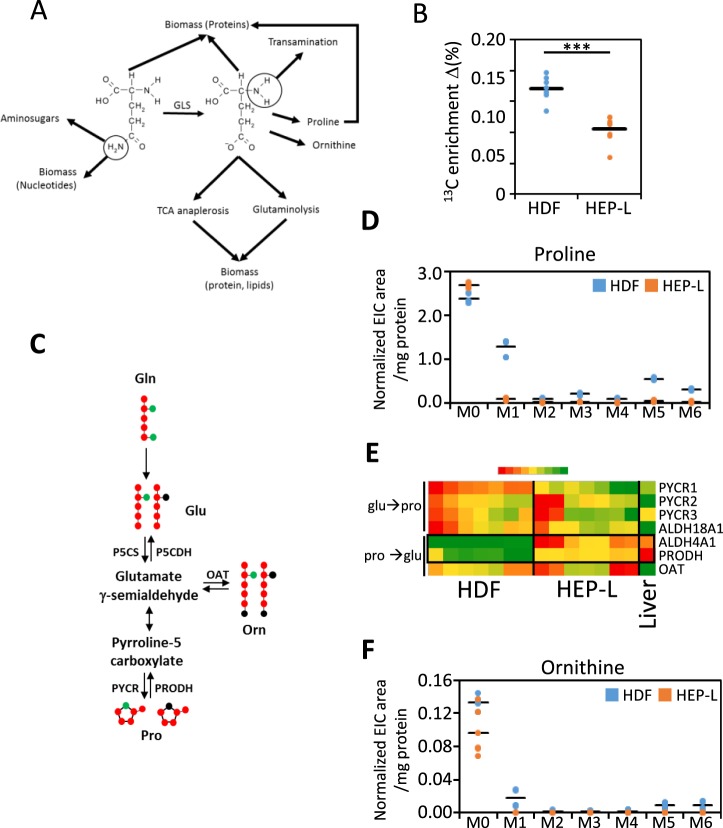
Higher glutamine utilization by HDF. (**A)** Diagram depicting the multiple glutamine/glutamate metabolic pathways. **(B)** ^13^C enrichment of cellular protein from HDF and HEP-L cells. Cell precipitates from methanol treatment were washed and ^13^C/^12^C ratio estimated by C/IRMS. ^13^C enrichment is defined as the difference between the percentage of ^13^C in sample and the natural abundance estimated in cells incubated without tracer (1.0799 ± 0.0023%; n = 8). Data is individually plotted (n = 8 per group); ***p < 0.001. **(C)** Diagram summarizing proline/ornithine biosynthetic pathway and the main isotopic metabolic transitions of ^13^C_5_^15^N_2_-Gln. P_5_CS: Δ^1^-pyrroline-5-carboxylate synthetase; P5CDH: Δ^1^-pyrroline-5-carboxylate dehydrogenase; PYCR: pyrroline-5-carboxylate reductase; PRODH: proline dehydrogenase; OAT: ornithine aminotransferase. Glutamate-γ-semialdehyde exists in equilibrium with pyrroline-5-carboxylate. **(D)** Cells were incubated with HMM media containing 2 mM ^13^C_5_^15^N_2_-Gln for _2_4 hours and intracellular levels of proline isotopologues determined by LC-MS and expressed as normalized extracted ion current (EIC) per mg of cellular protein. Values are individually plotted. N = 6 per group. **(E)** Heatmap representation of mRNA levels related to proline/ornithine biosynthesis determined by qRT-PCR in HDF and HEP-L cells and plotted as relative gene expression (Liver = 1). Numerical data provided in Table S6. N = 7. **(F)** Cells were incubated with HMM media containing 2 mM ^13^C_5_^15^N_2_-Gln for _2_4 hours and intracellular levels of ornithine isotopologues determined by LC-MS and expressed as normalized extracted ion current (EIC) per mg of cellular protein. Values are individually plotted. N = 6 per group. ***p < 0.001.

Glutamine metabolic pathways are summarized in Fig. 4A. To assess glutamine´s contribution to cellular protein, we quantified ^13^C isotopic enrichment in methanol precipitated cellular protein using combustion/isotope-ratio mass spectrometry (C/IRMS). HDF incorporated 70% more carbon-13 when incubated for 24 hours with ^13^C_5_^15^N_2_-Gln (Fig. 4B). Since, HDF and HEP-L cells are quiescent (Supplementary Fig. S5), this result cannot be attributed to higher proliferation rates. This result was not totally unexpected, since 58% of glutamine uptaken by HEP-L cells is secreted back to the media as glutamate. Thus, only 42% of glutamine (1.37 micromol/mg of protein*day compared to 1.79 micromol/mg of protein*day in HDF) is actually used in HEP-L cells for cellular metabolic processes apart from glutamine to glutamate deamidation and secretion.

De novo synthesis of proline, a major aminoacid in extracellular matrix proteins, was 14-fold higher in HDF (Fig. 4C,D). Upregulation of the catabolic enzymes ALDH4A1 (Δ^1^-pyrroline-5-carboxylate dehydrogenase; P5CDH) and PRODH (proline dehydrogenase) in HEP-L cells can explain this metabolic difference (Fig. 4E). This transcriptional change also explains the low production of labelled ornithine in HEP-L cells, given that both enzymes are shared in proline and ornithine synthesis pathway (Fig. 4F). Enhanced proline and ornithine catabolism in HEP-L cells are probably accounting for the presence of 15% non-glutamine, non-glucose derived glutamate in HEP-L cells supernatant^14^, since HMM media contains unlabeled ornithine and proline (100 and 30 mg/L respectively).

### Reductive glutamine metabolism is increased in HEP-L cells

**Figure 5:**
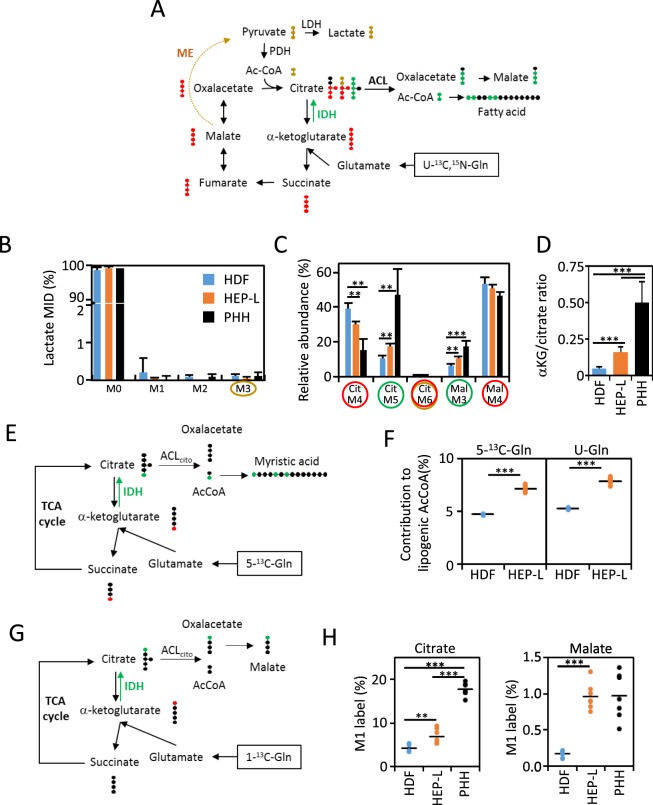
Increased glutamine reductive carboxylation in HEP-L cells. (**A)** Diagram of ^13^C labeling using U-^13^C,^15^N-Gln as the tracer. ^13^C_5_^15^N_2_-Gln enters the TCA cycle after deamination to ^13^C_5_-α-ketoglutarate. ^13^C_5_-α-ketoglutarate can be further metabolized via forward cycling to ^13^C_4_-succinate, ^13^C_4_-malate and so on (red). ^13^C_5_-α-ketoglutarate can also generate ^13^C_5_-citrate by reductive carboxylation initiated by reverse cycling catalysis of isocitrate dehydrogenase (IDH; green). As an external TCA pathway, ^13^C_4_-malate can be decarboxylated to ^13^C_3_-pyruvate by malic enzyme (ME; golden) that will ultimately produce ^13^C_3_-lactate or ^13^C_2_-acetyl-CoA. Citrate can be shuttled to the cytoplasm and produce oxaloacetate and lipogenic acetyl-CoA via ATP-citrate lyase. LDH: lactate dehydrogenase; PDH: pyruvate dehydrogenase; ACL: ATP citrate lyase. **(B)** Mass Isotopomer Distribution (MID) of intracellular lactate from cells incubated with ^13^C_5_^15^N_2_-Gln. **(C)** Relative percentages of intracellular selected labeled citrate and malate isotopologues in cells incubated with HMM media containing 2 mM ^13^C_5_^15^N_2_-Gln. **(D)** αKG to citrate ratios in HDF, HEP-L cells and PHH from cells incubated with unlabeled HMM media. Data in panels B, C and D is represented as mean ± s.d. from a total of 15 replicates per group (HDF and HEP-L cells) and 7 replicates from two experiments/donors (PHH). **(E)** Diagram summarizing isotopic metabolic transitions of 5-^13^C-Gln. Carbon-13 label is ultimately transferred to fatty acids after reductive carboxylation. **(F)** Contribution of 5-^13^C-Gln and ^13^C_5_^15^N_2_-Gln to lipogenic acetil-CoA. Values are individually plotted. N = 7 per group. **(G)** Diagram summarizing isotopic metabolic transitions of 1-^13^C-Gln. Carbon-13 label is ultimately transferred to citrate and malate after reductive carboxylation. **(H)** Intracellular percentage of ^13^C_1_-citrate and ^13^C_1_-malate (M1) in cells incubated with HMM media containing 2 mM 1-^13^C-Gln. Values are individually plotted. N = 7 per group. **p < 0.005; ***p < 0.001.

Glutamine can contribute carbon to lipogenic acetyl-CoA through glutaminolysis and reductive carboxylation. In glutaminolysis, ^13^C_5_^15^N_2_-Gln-derived ^13^C_5_-α-ketoglutarate generates ^13^C_4_-succinate, ^13^C_4_-fumarate and ^13^C_4_-malate isotopologues by forward cycling in TCA cycle (Fig. 5A). Decarboxylation of ^13^C_4_-malate via malic enzyme produces ^13^C_3_-pyruvate, which may give rise to ^13^C_3_-lactate by LDH or ^13^C_2_-acetyl-CoA by PDH. Using ^13^C_3_-lactate levels as a readout, we show that glutaminolysis pathway is of minor relevance in our experimental conditions (Fig. 5B), something expected for terminally differentiated cells^15^ as well as non-diving cells^16^. Moreover, condensation of ^13^C_2_-acetyl-CoA with ^13^C_4_-oxalacetate would produce ^13^C_6_-citrate, which is hardly detected (Fig. 5C). Alternatively, citrate can also be produced by reductive carboxylation of α-KG, which will ultimately render lipogenic acetyl-CoA by cytoplasmic ATP-citrate lyase (ACL) (Fig. 5A). We found higher contribution of ^13^C_5_-citrate and ^13^C_3_-malate, both suggestive of greater reductive carboxylation in HEP-L cells. In fact, α-KG to citrate ratio, a readout of reductive glutamine metabolism^17^ suggests that reductive carboxylation is approximately 2-3-fold and 10-fold more active in HEP-L cells and PHH respectively (Fig. 5D).

To definitively assess the contribution of glutamine to lipogenic acetyl-CoA through reductive or oxidative glutamine metabolism, we used ^13^C_5_^15^N_2_-Gln and 5-^13^C-Gln tracers and performed isotopomer spectral analysis^18,19^. Incubation with 5-^13^C-Gln allows to determine the contribution of glutamine to lipids by reducing carboxylation (Fig. 5E). Both cell types generated labelled myristic acid (C14:0), although HEP-L cells rate was 53% higher (Fig. 5F). When the same analysis was performed using ^13^C_5_^15^N_2_-Gln as a tracer to estimate the total contribution of glutamine to fatty acid synthesis, no difference was found, indicating that reductive carboxylation is the primary route for glutamine incorporation into fatty acids. Finally, we quantified the retention of isotope label in citrate and malate pools from cells incubated with 1-^13^C-Gln (Fig. 5G). HEP-L cells accumulated more 1-^13^C isotopologues. However, we found differences between label retention in citrate and malate and also between HEP-L cells and primary cultured human hepatocytes (Fig. 5H).

## Discussion

Direct conversion of human fibroblasts to HEP-L cells can be achieved by expression of a specific set of transcription factors^2^. HEP-L cells have been characterized by gene expression profiling and limited functional assays, such as CYP450 activity, glycogen storage (PAS staining) or ac-LDL intake. However, metabolic reprogramming of HEP-L cells (switch between fibroblastic and hepatic metabolic features) has not been covered in these studies. In this paper, we have addressed changes in glutamine/glutamate metabolic pathways in reprogrammed cells and the transcriptomic changes underneath. HEP-L cells acquire a high capacity to deamidate glutamine into glutamate and secrete it into the media, diverting glutamate from other metabolic pathways; moreover glutamate-ammonia ligation to glutamine is reduced. Incorporation of glutamine/glutamate carbon moiety to cell protein is reduced, as well as the synthesis of proline from glutamate. However, HEP-L cells significantly increase glutamine anaplerosis into fatty acids by reductive carboxylation. All this characteristics are in agreement with the metabolism of primary cultured human hepatocytes as shown in this work.

Glutamine/glutamate metabolism is heterogeneous in the liver. This is accomplished by drastic differences in periportal to perivenous expression of glutamine/glutamate transporters, phosphate-activated glutaminase (GLS2) and glutamine synthase (GLUL)^9,13^. Periportal hepatocytes uptake glutamine from the portal vein, deamidate it and secrete large amounts of glutamate while keeping glutamine synthesis very low^11^. HEP-L cells also perform this glutamate-secreting pathway by de novo expression of liver-specific glutaminase (GLS2), not inhibited by glutamate concentration^8,9^, at levels comparable to human liver (90%), remodeling of glutamate transport expression and low levels of glutamine synthase (GLUL) a classical perivenous hepatocyte marker^13^. A shift in the intracellular-extracellular concentration gradient of glutamate might also be critical for glutamate flux reversal (Supplementary Fig. S6).

Glutamine metabolic pathways participate in proteins, lipids and sugar metabolism. We found that HEP-L cells reduce the utilization of glutamine for intracellular metabolism when compared to HDF. Among the different pathways, we detected reduced incorporation of glutamine carbon moiety to cellular protein and reduction of proline synthesis. Surprisingly, glutamine anaplerosis into fatty acids by reductive carboxylation (Gln→aKG→Cit→Acetyl-CoA_lipo_) was higher in HEP-L cells.

According to our analysis, HDF reprogramming to HEP-L cells involves a metabolic rewiring that leads to the loss of metabolic pathways characteristic of HDF and the acquisition of metabolic pathways present in hepatocytes in concurrence with changes in the transcription of metabolic-related genes. Fibroblast is one of the most common collagen-producing cell in the body. Collagen is composed by 28% of proline/hydroxyproline^6^. Proline is synthesized in the cell from glutamate^20^. HEP-L cells hardly accumulated labeled proline despite containing 3-fold higher glutamate concentration (Supplementary Fig. S6) and we found that expression of genes involved in degradation of proline to glutamate were the most probable cause of this change. Yet, metabolic differences exist between HEP-L cells and PHH. Contribution of glutaminase activity to glutamate pool is several fold greater in HEP-L cells. Also, the importance of reductive carboxylation in glutamine anaplerosis is of higher relevance in PHH based on several parameters, i.e. the levels of citrate M5 and malate M3 in cells incubated with ^13^C_5_^15^N_2_-Gln, the ratio αKG/citrate in cells incubated with unlabeled media and the levels of citrate M1 in cells incubated with 1-^13^C-Gln. It is interesting to note that we found important differences between malate and citrate M1 abundance in the latter incubations.

Reductive carboxylation of glutamine-glutamate derived α-ketoglutarate was initially described as an alternate pathway in rat liver^21^. In their tracing experiments, 5-14C-glutamate incorporated to fatty acids (10%). Ulterior papers confirmed the original results in liver and elaborated a sophisticated model based on the reversibility of isocitrate dehydrogenase^12,22–24^. Briefly, using 13 C glutamate tracers, a striking 10.8% M5 citrate when M5 glutamate was perfused in rat liver. In fact, M5 enrichment of citrate amounts to 45% of the M5 enrichment of α-ketoglutarate. The authors concluded that the formation of M5 citrate can only be explained by the reversal of the isocitrate dehydrogenase reaction. Later studies concluded that reversion of isocitrate dehydrogenase reaction would provide cytosolic acetyl-CoA from mitochondrial α-ketoglutarate for fatty acid synthesis. Thus, the reversal of the isocitrate dehydrogenase reaction should be included in isotope labeling models of the citric acid cycle. Reductive carboxylation was later observed in heart and brown adipocytes^25^. The role of reductive carboxylation in liver, heart or adipose tissue, is to maintain lipogenesis under hypoxia, when acetyl-CoA supply from glucose-derived oxidation might be limited^26^. We interpret the increased capacity of HEP-L cells to divert glutamine via reductive carboxylation to FA synthesis compared to HDF as an important metabolic reprogramming into the hepatocyte cell lineage supporting a metabolic rewiring of glutamine-glutamate metabolism.

In summary, we found major changes in glutamine/glutamate metabolism in reprogrammed HEP-L cells compatible with hepatocyte function and validated using freshly isolated primary cultured human hepatocytes. In particular, some of these changes are suggestive of periportal hepatocyte function i.e. intense deamidation of glutamine, secretion of large amounts of glutamate^27^, low glutamine synthesis, high expression of *GLS2* mRNA and down-regulation of *GLUL* mRNA. We also found the loss of active proline synthesis characteristic of HDF. All these changes are in agreement with reprogramming into HEP-L cells. Nevertheless, metabolite pathways differences exist between HEP-L cells and mature hepatocytes that are probably indicating uncomplete reprogramming. In fact, our conclusions are limited by the fact that HEP-L cells cells generated in this study do not display a complete mature phenotype. Expression of genes such as CYP2C9, CYP2C19, CYP2D6, ARG1, OTC, CPS1, PXR, CAR, NURR1 or ESR1^28^ are hardly expressed compared to hepatocytes (data not shown). Implementation of recently improved protocols^29^, will probably bring metabolic function in HEP-L cells closer to PHH. In this sense, future metabolic profiling of cells in the process of reprogramming, may also help identify metabolic barriers that hamper HEP-L cells reprogramming and design improved cell media to facilitate a complete reprogramming into hepatocytes.

## Methods

All methods were carried out in accordance with relevant guidelines and regulations. All experimental protocols were approved by the Instituto de Investigación Sanitaria la Fe.

### Plasmids and Lentivirus Generation

The lentiviral vector pHIV-EGFP-FOXA3 was obtained by PCR-cloning human FOXA3 cDNA into BamHI-XbaI restriction sites in pHIV-EGFP^30^ (Addgene #21373). The lentiviral vector pHIV-dTOM-HNF4A was obtained by PCR-cloning human HNF4A cDNA into EcoRI restriction site in pHIV-dTomato (Addgene #21374). The lentiviral vector pHIV-RFP was obtained by substituting EGFP by synthesized RFP657 sequence^31^ in pHIV-EGFP vector (GeneArt®; ThermoFisher). The lentiviral vector pHIV-RFP-HNF1A was obtained by cloning human HNF1A cDNA into pHIV-RFP (GeneArt®; ThermoFisher). All constructs were verified by sequencing. Lentivirus were generated in 293 T cells by cotransfection of pHIV vector with pPAX2 and pMD2.G in 10:7.5:5 ratio. Lentivirus were collected and concentrated using Lenti-X concentrator (Clontech).

### Cell culture and imaging

Human dermal fibroblasts were purchased from ATCC® (CRL-2429). Human dermal fibroblasts were reprogrammed as described in detail elsewhere^2^. Briefly, cells were seeded on collagen coated plates and infected with equal amounts of lentiviral vectors encoding HNF4A, HNF1A and FOXA3 or the corresponding empty vectors (control; HDF). Cells were kept in HFM media: DMEM/F12 w/o glutamine, w/o glucose (Biowest, Nuaillé, France) supplemented with glutamine (2 mM), glucose (3.15 g/L), 10% Fetal bovine serum, 0.1 mM β-mercaptoethanol, 1x MEM Non-Essential Amino Acids Solution and 4 ng/ml bFGF) for 48 hours and then switched to HMM media: DMEM/F12 w/o glutamine, w/o glucose supplemented with glutamine (2 mM), glucose (3.15 g/L), 0.544 mg/L ZnCl_2_, 0.75 mg/L ZnSO_4_·7H_2_O, 0.2 mg/L, CuSO_4_·5H_2_O, 0.025 mg/L MnSO_4_, 2 g/L Bovine serum albumin, 0.1 g/L Ornithine, 0.03 g/L Proline, 0.61 g/L Nicotinamide, 1X Insulin-transferrin-sodium selenite media supplement, 40 ng/ml TGFα, 40 ng/ml EGF, 10 μM dexamethasone and 1% fetal bovine serum) for 10 additional days. All media was purchased from ThermoFisher (Waltham, MA, USA). Growth factors were purchased to Peprotech (London, UK). Cells were maintained at 37 °C with 5% CO_2_ and were regularly examined with an Olympus CKX41 microscope.

Human liver samples were obtained in agreement with the rules of the hospital’s Ethics Committee. Informed consent was obtained from all donors. Patients had no known liver pathology nor did they receive medication during the weeks prior to surgery. None of the patients were habitual consumers of alcohol or other drugs. Human hepatocytes were isolated from 2 liver biopsies (<5 g), using a two-step collagenase perfusion technique^32^. Hepatocytes were seeded on fibronectin/collagen-type I-coated (Sigma-Aldrich, Madrid, Spain) dishes and cultured with Ham’s F-12/Williams (1:1) medium (Gibco BRL, Paisley, Scotland). Seeded hepatocytes were allowed to settle for 6 hours before experiments were initiated (Table S3).

### qRT-PCR, immunofluorescence, PAS staining, Indocyanine green transport and ELISA

qRT-PCR, immunofluorescence, PAS staining and Indocyanine green transport were performed as previously described^33^. Control Liver RNA sample was obtained by combining human liver total RNA from five donors (Table S4). The antibodies used in this study are described in Table S5. Fluorescence images were taken in Olympus FV1000 confocal mounted on an IX81 inverted microscope.

To determine the presence of human albumin in cell media and mice sera, we used a human Albumin ELISA Quantitation Set (Bethyl Laboratory; Montgomery, TX, USA) according to the manufacturer´s instructions. Reference value (primary cultured human hepatocytes) were obtained from previous data from our group^34^.

### *In vivo* transplantation

Transplantation of HEP-L cells in male CB17/Icr-Prkdc scid/Crl mice was done as previously described^35^. Animals were acquired from Charles River Laboratories (Willmington, MA, USA) and housed at the animal facilities of the Instituto de Investigación Sanitaria La Fe. All experiments were performed in accordance with relevant guidelines and regulations and approved by the Institutional Animal Ethics Committee of the Instituto de Investigación Sanitaria La Fe and Generalitat Valenciana (reference number IP.RBM.#6A-3-2015). Briefly, three hours after the injection of 300 mg/kg of APAP, mice were anaesthetized with a sevoflurane/O_2_ mixture and the lower pole of the spleen was exposed. Animals received an intrasplenic injection of 10^6^ HEP-L cells^LT^ cells in 200 μl of PBS within seconds. The control mice, which had also received APAP treatment, received an intrasplenic injection of PBS. Thirty days after infusion, mice were sacrificed under anaesthesia (sevoflurane/O_2_ mixture). Blood was collected and serum aliquots were protected from light and stored at −80 °C until analysis.

### Solvents and chemicals for metabolite analysis

LC-MS grade methanol (MeOH) formic acid (FA) and acetonitrile (ACN) were from Fisher Scientific (Pittsburgh, PA, USA). Water was of ultrapure grade (EMD Millipore Co., Billerica, MA, USA). Deuterated internal standards (IS) D5-Glutamic acid, D5-Phenylalanine and D4-Succinic acid were from Cambridge Isotope Laboratories (Tewksbury, MA, USA). [U-^13^C_5_^15^N_2_]-glutamine, [5-^13^C]-glutamine and [1-^13^C]-glutamine were from Cortecnet (Paris, France). Glutamine, glutamic acid, [U-^13^C_6_]-glucose, ornithine, fumaric acid, aspartic acid, alpha-ketoglutaric acid, alanine and lactic acid were from Sigma (Sigma-Aldrich, St. Louis, MO, USA). Commercial negative/positive calibration and reference (lock masses) solutions for the MS device were from Agilent Technologies (Santa Clara, CA, USA).

### Isotopic labelling and sample processing

HDF cultured on collagen coated 12w plates were infected with lentiviral vectors expressing HNF4, HNF1A and FOXA3. After 11 days, glutamine or glucose in HMM media was substituted with isotope-labelled compounds at the same final concentration. Cell media was collected after 24 hours and immediately frozen in liquid N_2_, and kept at −80 °C until analysis. Before analysis, samples were diluted 1/100 with water containing D4-Succinic acid, D5-Glutamic and D5-Phenylalanine as internal standards (IS; final concentration 2ppm) and filtered through a modified PES 3 K molecular exclusion filter (VWR; Radnor, PA, USA). Cell monolayers were washed three times with 1 mL of cold PBS and metabolites extracted immediately by scraping the cells with 600 µL of a methanol/water (4:1) solution at −20 °C. The cell extract was transferred to a clean tube and immediately frozen in liquid N_2_, and kept at −80 °C for at least 24 hours. When ready for analysis, samples were centrifuged at 12000 rpm for 15 min at 4 °C. The supernatant was transferred to a clean tube, IS added and dried under vacuum; the dry residue was stored at −80 °C until analysis. Before analysis, residues were resuspended in 50 µL of water. Pellet was resuspended in 600 μL NaOH 0.5 M and protein quantified by Lowry in a small aliquote. The rest of the pellet was washed twice with cold methanol/water (4:1) solution to decrease sodium concentration and ^13^C enrichment determined by C/IRMS (Combustion/Isotope Ratio Mass Spectrometry) using a stable isotope ratio mass spectrometer (Thermo Finnigan^TM^ MAT253) coupled to an elemental analyzer (Carlo Erba EA1108) in Servizos de Apoio a Investigacion (SAI, Universidade da Coruña).

### Liquid chromatography high-resolution mass spectrometry (LC-HRMS) analysis

Chromatographic analysis was performed on an Agilent 1290 Infinity II (Agilent Technologies, Santa Clara, CA, USA) HPLC system equipped with a quaternary pump, vacuum degasser and an autosampler with a temperature controller. Chromatographic separation of metabolites was achieved on a 150 mm × 2.1 mm, 4 µm particle size Synergi-Hydro C18 column (Phenomenex Inc, Torrance, CA, USA) with the following separation conditions: solvent A, water/FA (99.8:0.2); solvent B, ACN; separation gradient, initially 1% B, held for 2 min and then linear 1–80% B in 8 min, washing with 98% B for 2 min and column equilibration with 1% B for 7 min; flow rate, 0.25 mL/min; injection volume range, 0.2–4.5 µl. Autosampler and column temperatures were set at 6 °C and 23 °C, respectively.

Mass spectrometry analysis was carried out by an Agilent 6550 Q-ToF (Agilent Technologies, Santa Clara, CA, USA) detector equipped with an electrospray (ESI) source with Jet Stream Technology. Column flow was conducted into the mass analyzer in the time range of 0.7–12 min diverting the rest of run time to waste. Isotopologue study of samples was separately performed in positive and negative ionization modes under full MS scan mode. Positive assignments were achieved through autoMS/MS analysis with exclusion and inclusion lists in the respective ionization mode. Shared MS conditions of analysis were: gas temp, 130 °C; drying gas, 14 L/min; nebulizer, 30 psig; sheath gas, 10 L/min; capillary voltage, 3500 V and 3000 V for positive and negative ionization modes, repectively; fragmentor, 380 V; octapole 1 RF, 400 V; isolation width, narrow (1.3 m/z); Nozzle voltage, 500 V funnel exit DC, funnel RF HP and funnel exit RF LP, 50, 150 and 60 V, respectively; lock masses, 121.0509/922.0098 and 119.0363/980.0164 for positive and negative, respectively; considered m/z range, 40–750; data acquisition, centroid mode.

Particular settings for full MS isotopologue analysis: acquisition rate, 1.5 scans/sec (cycle time, 0.666 sec). The autoMS/MS analyses merged full MS with MS/MS experiments with settings: acquisition rate for both scan events, 4 scans/sec (cycle time, 1.1 sec); collision induced dissociation (CID) energies, 10 and 20; Max precursors per MS/MS cycle, 3; Active MS/MS exclusion, after 2 scans during 0.3 min; isotope mode, off; Precursor threshold, 3000 counts; exclusion list, included lock masses and those from a blank injection; inclusion list; included m/zs from low-abundance metabolites in samples.

Before sample analysis, the MS device was tuned and calibrated in low mass range and high resolution mode (4 GHz). Considered mass tolerance for full MS and MS/MS analyses for data processing was 10 ppm.

### LC-MS data processing

Untargeted cell media metabolic profiling was performed by analyzing processed media samples from cells incubated with unlabeled nutrients with the Molecular Feature Extraction wizard of the Profinder B.08.00 program. Molecular features (characterized by exact mass and retention time) with MFE score above 95% and present in all samples of at least one group (HDF or HEP-L cells) were selected for statistical analysis.

Positive assignments were manually achieved by the comparison of the MS/MS breakdown profiles of metabolites from samples with those from either commercial standards and detailed in the Metlin database (https://metlin.scripps.edu). Two in-house libraries listing the identified compounds were built to carry out automated data processing. Automated peak integration of unlabeled and labeled metabolites from samples was carried out by the Targeted Feature Extraction wizard of the Profinder B.08.00 program. In this case, there was loaded a customized library detailing the retention time and neutral unlabeled/labeled masses of identified metabolites considering all the isotopologue combinations (from [M0] to [Mn]), while setting isotope abundance and spacing scores to 0%. Mass and retention time tolerances used by Profinder analysis were 10 ppm and 0.2 min, respectively. Isotopologues abundance were corrected for the presence of naturally occurring isotopes using FLUXFIX software^36^.

Absolute quantification of glutamic acid and glutamine isotopologues was carried out through their respective relative response factors using D5-glutamic and D4-succinic acids as IS, respectively. The rest of metabolites considered in this research were normalized and relatively quantified through the integrated peak area ratios of analytes and their respective IS. Absolute intracellular metabolite concentration was determined dividing the amount of metabolite in the cell extract/plate expressed as milimoles by total cellular volume determined in parallel plates (average cell volume x plate cell number). Alternatively, LC/MS data was normalized by mg of protein in the extracted cell pellet.

### Fatty acid analysis

Lipidome was extracted and total lipids were saponified as described previously^37^. Chromatographic analysis was performed on an an Acquity Ultra Performance LC system (Waters, Milford, MA, USA) equipped with an Acquity UPLC BEH C18 column (100 × 2.1 mm; 1.7 µm) (Waters, Milford, MA, USA) as described^38^. Mass spectrometry analysis was carried out by a Synapt G2-Si Q-TOF (Waters, Milford, MA, USA).a, CA, USA) detector equipped with an electrospray (ESI) source with Jet Stream Technology. Data analysis was performed using LipidMS package (https://CRAN.R-project.org/package=LipidMS). Labelling of fatty acids for isotopomer spectral analysis (ISA) was conducted over 2 days of culture with medium change after the first day to prevent tracer/nutrient depletion. ISA was performed using the simple network described in^18^. Uncorrected ^13^C mass isotopomer distributions were fitted to the model equations and the tracer enrichment in lipogenic acetyl-CoA (D value) and percentage of newly synthesized lipids and de novo lipogenesis, g(t) were estimated by GRG nonlinear evolutionary method using Solver add-in in Excel^39^.

### NMR analysis

Samples were placed on ice and allowed to thaw for 20 min. 500 µl of medium was added to 100 µl of phosphate buffer (100 mM Na2HPO4 pH 7.4, in 100% D2O) containing 15 mM 3-(trimethylsilyl)propionic −2,2,3,3-d4 acid sodium salt (TSP), as internal standard. The samples were vortexed and transferred into a 5 mm NMR tube. Samples were analyzed on a Bruker Ultrashield Plus 600 MHz spectrometer equipped with a 5 mm TCI cold probe. 13 C experiments were acquired with a 29761 Hz spectral width, 32768 data points, acquisition time of 0.55 s, relaxation delay of 60 s and 256 scans at 27 °C. Spectra were processed with exponential line broadening to 3 Hz and zero filling to 65536 points. Multiplicity Heteronuclear Single Quantum Correlations (HSQC) were performed for representative samples for signal assignment. For these experiments, 256 t1 increments were used and 32 transients were collected. The relaxation delays were set to 1.5 s and the experiments were acquired in the phase-sensitive mode. Following Fourier transformation, 1D spectra were manually phased, baseline corrected and referenced to the TSP peak (0.00 ppm) using MestReNova 8.1. Metabolite identities were assigned by comparison to reference values for chemical shift and multiplicity. Quantification was carried out with the Eretic Signal (Bruker Biospin). Metabolite signals were integrated with MestreNova 8.1 using the GSD deconvolution option.

### Statistical analysis

The number of experiments (n) throughout the paper refers to the number of independent biological replicates (cell plates, animals…). The infection was performed using lentivirus generated in three time-separated batches. Sample size was estimated using Monte Carlo Simulations. Based on mean values and standard deviations from preliminary experiments a sample size of 7 per group was needed to achieve a statistical power of 80% at the standard significance level of alpha = 0.05. When needed the sample size was increased to 15. Primary cultured human hepatocytes used for metabolic studies were derived from two donors (a total of 7 replicates distributed in 3 and 4 cell plates from each donor). Descriptive statistics (mean ± s.d.) are used for experiments with n ≥ 10. When n < 10, individual points are plotted. Statistical differences were calculated using two-tailed Student’s t-test for unpaired samples (also known as Welch’s t-test). Normal distribution was confirmed using the Shappiro-Wilk test. Volcano plot was generated using Metaboanalyst software (www.metaboanalyst.ca). Data related to every metabolite-related determination, was acquired by an investigator blinded to the group allocation since all samples were number-coded. Data analysis was performed by another investigator.

## Supplementary information

Supplementary information

## Data Availability

The datasets generated during and/or analysed during the current study are available from the corresponding author on reasonable request.

## References

[1] Du Y (2014). Human hepatocytes with drug metabolic function induced from fibroblasts by lineage reprogramming. Cell Stem Cell.

[2] Huang P (2014). Direct reprogramming of human fibroblasts to functional and expandable hepatocytes. Cell Stem Cell.

[3] Nakamori D, Akamine H, Takayama K, Sakurai F, Mizuguchi H (2017). Direct conversion of human fibroblasts into hepatocyte-like cells by ATF5, PROX1, FOXA2, FOXA3, and HNF4A transduction. Sci. Rep..

[4] Simeonov KP, Uppal H (2014). Direct reprogramming of human fibroblasts to hepatocyte-like cells by synthetic modified mRNAs. PloS One.

[5] Lemons JMS (2010). Quiescent fibroblasts exhibit high metabolic activity. PLoS Biol..

[6] Eastoe JE (1955). The amino acid composition of mammalian collagen and gelatin. Biochem. J..

[7] Gebhardt R (1992). Metabolic zonation of the liver: regulation and implications for liver function. Pharmacol. Ther..

[8] Krebs HA (1935). Metabolism of amino-acids: The synthesis of glutamine from glutamic acid and ammonia, and the enzymic hydrolysis of glutamine in animal tissues. Biochem. J..

[9] Watford M (2000). Glutamine and glutamate metabolism across the liver sinusoid. J. Nutr..

[10] Häussinger D, Gerok W (1983). Hepatocyte heterogeneity in glutamate uptake by isolated perfused rat liver. Eur. J. Biochem..

[11] Hediger MA, Welbourne TC (1999). Introduction: glutamate transport, metabolism, and physiological responses. Am. J. Physiol..

[12] Des Rosiers C (1995). Isotopomer analysis of citric acid cycle and gluconeogenesis in rat liver reversibility of isocitrate dehydrogenase and involvement of ATP-citrate lyase in gluconeogenesis. J. Biol. Chem..

[13] Halpern KB (2017). Single-cell spatial reconstruction reveals global division of labour in the mammalian liver. Nature.

[14] Brosnan ME, Brosnan JT (2009). Hepatic glutamate metabolism: a tale of 2 hepatocytes. Am. J. Clin. Nutr..

[15] DeBerardinis RJ (2007). Beyond aerobic glycolysis: transformed cells can engage in glutamine metabolism that exceeds the requirement for protein and nucleotide synthesis. Proc Natl Acad Sci U A.

[16] Altman BJ, Dang CV (2012). Normal and cancer cell metabolism: lymphocytes and lymphoma. FEBS J..

[17] Fendt S-M (2013). Reductive glutamine metabolism is a function of the α-ketoglutarate to citrate ratio in cells. Nat. Commun..

[18] Kelleher, J. K. & Nickol, G. B. Isotopomer spectral analysis: utilizing nonlinear models in isotopic flux studies. in *Methods in enzymology* **561**, 303–330 (Elsevier, 2015). 10.1016/bs.mie.2015.06.039 26358909

[19] Metallo CM (2012). Reductive glutamine metabolism by IDH1 mediates lipogenesis under hypoxia. Nature.

[20] Barbul A (2008). Proline precursors to sustain Mammalian collagen synthesis. J. Nutr..

[21] D’Adamo AF, Haft DE (1965). An alternative pathway of alpha-ketoglutarate catabolism in the isolated, perfused rat liver. I. Studies with DL-Glutamate-2- and −5-14C. J. Biol. Chem..

[22] Des Rosiers C, Fernandez CA, David F, Brunengraber H (1994). Reversibility of the mitochondrial isocitrate dehydrogenase reaction in the perfused rat liver. Evidence from isotopomer analysis of citric acid cycle intermediates. J. Biol. Chem..

[23] Fernandez CA, Des Rosiers C (1995). Modeling of liver citric acid cycle and gluconeogenesis based on 13C mass isotopomer distribution analysis of intermediates. J. Biol. Chem..

[24] Wanders RJ, van Doorn HE, Tager JM (1981). The energy-linked transhydrogenase in rat liver in relation to the reductive carboxylation of 2-oxoglutarate. Eur. J. Biochem..

[25] Yoo H, Antoniewicz MR, Stephanopoulos G, Kelleher JK (2008). Quantifying reductive carboxylation flux of glutamine to lipid in a brown adipocyte cell line. J. Biol. Chem..

[26] Metallo CM (2011). Reductive glutamine metabolism by IDH1 mediates lipogenesis under hypoxia. Nature.

[27] Jang C (2019). Metabolite Exchange between Mammalian Organs Quantified in Pigs. Cell Metab..

[28] Zabulica M (2019). Guide to the Assessment of Mature Liver Gene Expression in Stem Cell-Derived Hepatocytes. Stem Cells Dev..

[29] Xie Bingqing, Sun Da, Du Yuanyuan, Jia Jun, Sun Shicheng, Xu Jun, Liu Yifang, Xiang Chengang, Chen Sitong, Xie Huangfan, Wang Qiming, Li Guangya, LYU Xuehui, Shen Hui, Li Shiyu, Wu Min, Zhang Xiaonan, Pu Yue, Xiang Kuanhui, Lai Weifeng, Du Peng, Yuan Zhenghong, Li Cheng, Shi Yan, Lu Shichun, Deng Hongkui (2019). A two-step lineage reprogramming strategy to generate functionally competent human hepatocytes from fibroblasts. Cell Research.

[30] Welm BE, Dijkgraaf GJP, Bledau AS, Welm AL, Werb Z (2008). Lentiviral transduction of mammary stem cells for analysis of gene function during development and cancer. Cell Stem Cell.

[31] Morozova KS (2010). Far-red fluorescent protein excitable with red lasers for flow cytometry and superresolution STED nanoscopy. Biophys. J..

[32] Donato MT (2008). Functional assessment of the quality of human hepatocyte preparations for cell transplantation. Cell Transplant..

[33] Serrano F (2016). Silencing of hepatic fate-conversion factors induce tumorigenesis in reprogrammed hepatic progenitor-like cells. Stem Cell Res. Ther..

[34] Tolosa L (2015). Human neonatal hepatocyte transplantation induces long-term rescue of unconjugated hyperbilirubinemia in the Gunn rat. Liver Transplant. Off. Publ. Am. Assoc. Study Liver Dis. Int. Liver Transplant. Soc..

[35] Tolosa L (2015). Transplantation of hESC-derived hepatocytes protects mice from liver injury. Stem Cell Res. Ther..

[36] Trefely S, Ashwell P, Snyder NW (2016). FluxFix: automatic isotopologue normalization for metabolic tracer analysis. BMC Bioinformatics.

[37] Kamphorst JJ, Fan J, Lu W, White E, Rabinowitz JD (2011). Liquid chromatography–high resolution mass spectrometry analysis of fatty acid metabolism. Anal. Chem..

[38] García-Cañaveras JC, Castell JV, Donato MT, Lahoz A (2016). A metabolomics cell-based approach for anticipating and investigating drug-induced liver injury. Sci. Rep..

[39] Kemmer G, Keller S (2010). Nonlinear least-squares data fitting in Excel spreadsheets. Nat. Protoc..

