# Supplementary material for: Glutamine/glutamate metabolism rewiring in reprogrammed human hepatocyte-like cells
Source: Sci Rep. 2019 Nov 29;9:17978. doi: 10.1038/s41598-019-54357-x (PMC6884617; doi:10.1038/s41598-019-54357-x)
Supplement: Supplementary file 1 — Supplementary information [file 41598_2019_54357_MOESM1_ESM.pdf]

**SUPPLEMENTARY INFORMATION**

# Glutamine/glutamate metabolism rewiring in reprogrammed human hepatocyte-like cells

**Maria Ballester[1], Enrique Sentandreu[1], Giovanna Luongo[1], Ramon Santamaria[1], Miguel Bolonio[1], Maria Isabel Alcoriza-Balaguer[3], Martina Palomino-Schätzlein[2], Antonio Pineda-Lucena[2], Jose Castell[1], Agustin Lahoz[3] and Roque Bort[1*]**

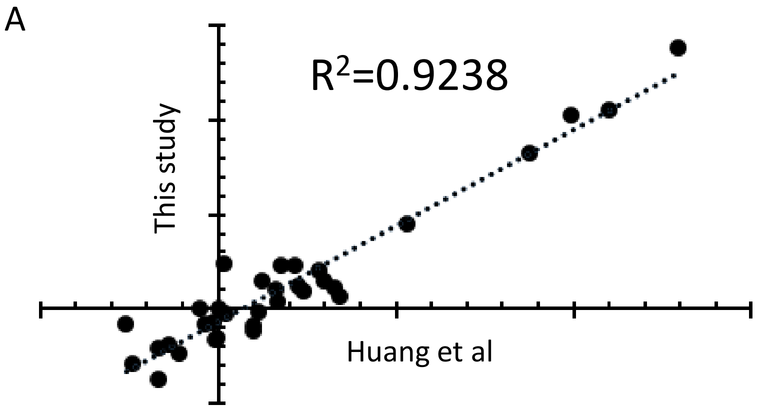
A
R$^2$=0.9238
This study
Huang et al

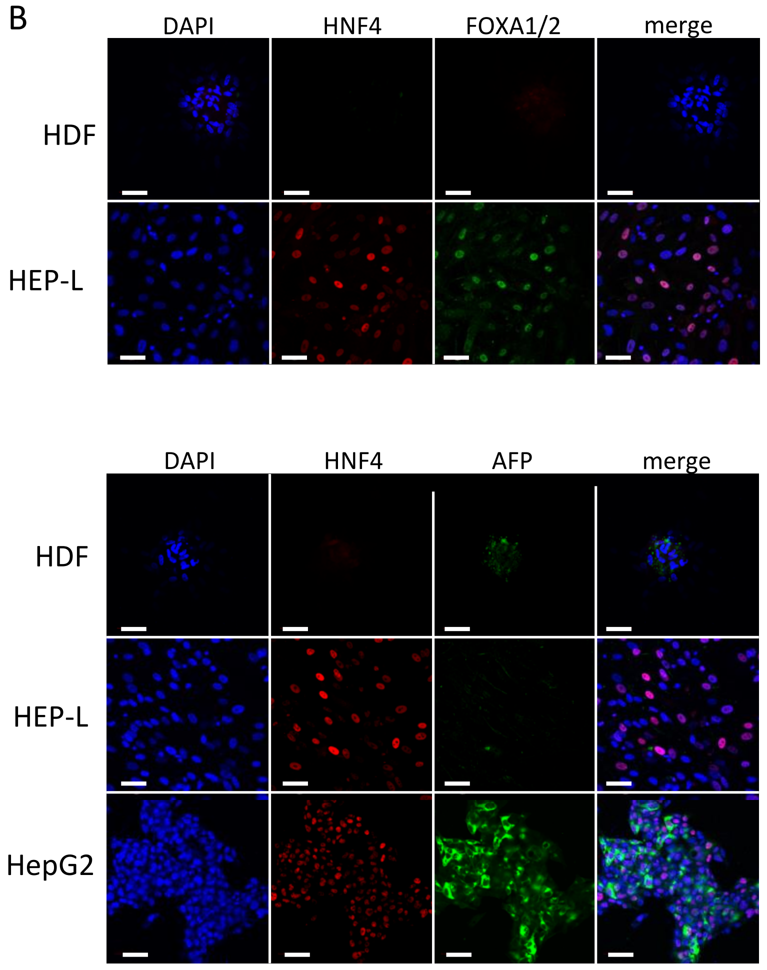
B
DAPI
HNF4
FOXA1/2
merge
HDF
HEP-L
DAPI
HNF4
AFP
merge
HDF
HEP-L
HepG2

Figure S1

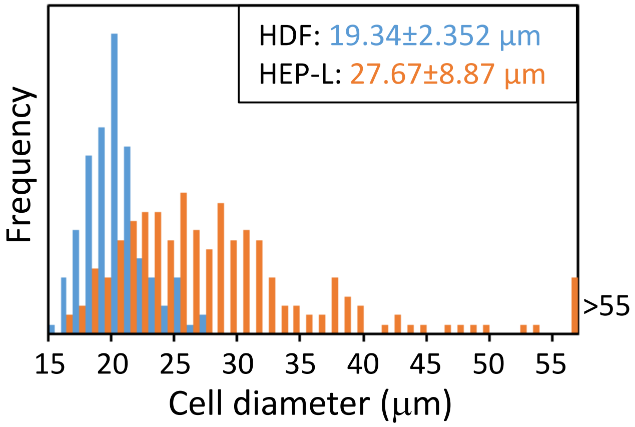


| |
|---|
| Cellular volume ($\frac{4}{3}\pi r^3$)<br>HDF: 3787±68 μm$^3$<br>HEP-L: 11098±365 μm$^3$ |
| Protein (ng/cell)<br>HDF: 1.31±0.31<br>HEP-L: 5.40±0.76 |
| Protein (μg/mm$^3$ of cell)<br>HDF: 330.42±79.40<br>HEP-L: 358.76±50.49 |

Figure S2

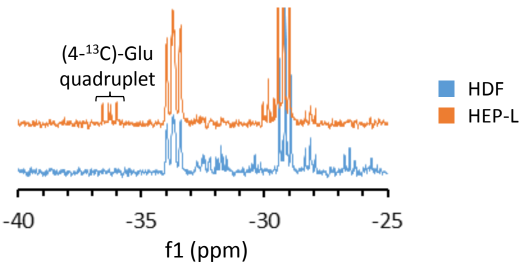


Figure S3

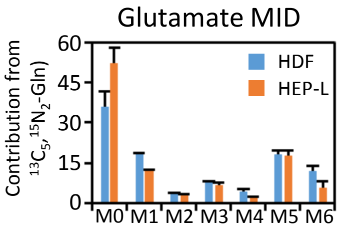

Glutamate MID
Contribution from
$^{13}C_5$, $^{15}N_2$-Gln)
60
45
30
15
0
HDF
HEP-L
M0
M1
M2
M3
M4
M5
M6


Figure S4

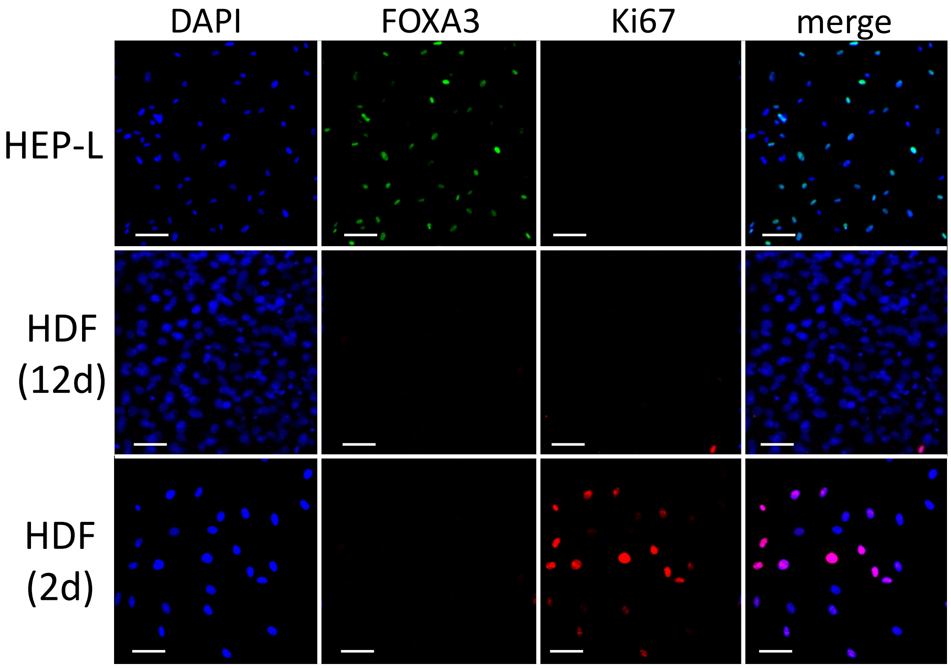


Figure S5

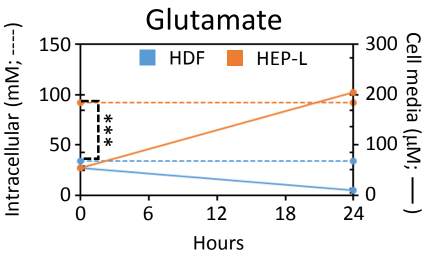
Glutamate
HDF
HEP-L
Intracellular (mM; ----)
Cell media (µM; —)
150
100
50
0
300
200
100
0
***
0
6
12
18
24
Hours

# Figure S6

**Supplementary Table 1.**

List of metabolites/features differentially concentrated in cell supernatant from HDF and HEP-L cells after 24 hours. Settings (www.metaboanalyst.ca): fold change>2 and FDR<0.005. Candidate formula was obtained using HMDB database (www.hmdb.ca) with an accuracy of 15ppm. Glutamate is in bold.

| Mass (neutral) | Rt (min) | Formula | Mass (neutral) | Rt (min) | Formula | Mass (neutral) | Rt (min) | Formula | Mass (neutral) | Rt (min) | Formula |
|---|---|---|---|---|---|---|---|---|---|---|---|
| 74.0365 | 4.9 | C3H6O2 | 220.9812 | 1.42 | | 308.1201 | 7.41 | C18H16N2O3 | 426.0901 | 1.66 | C13H22N4O8S2<br>C22H18O9 |
| 88.0161 | 1.84<br>2.26 | C3H4O3 | 221.9966 | 1.85 | C5H6N2O6S | 311.9743 | 3.53 | | 429.995 | 3.53 | C22H22ClNO6 |
| 103.064 | 1.42 | C4H9NO2 | 233.9964 | 3.53 | | 312.0642 | 4.9 | C17H12O6 | 431.1136 | 7.45 | C15H21N5O8S |
| 104.0474 | 4.9 | C4H8O3 | 234.0056 | 4.9 | C8H5F3N2OS | 316.0253 | 4.9 | C12H12O8S<br>C15H8O8 | 439.1425 | 7.27 | C19H25N3O7S<br>C16H29N3O7S2<br>C21H26ClNO7 |
| 112.0161 | 3.52 | C5H4O3 | 234.0341 | 5.42 | C8H10O8 | 327.1207 | 1.42 | | 440.0844 | 7.4 | |
| 118.0628 | 5.43<br>5.79 6.1 | C5H10O3 | 244.0705 | 2.66 | C9H12N2O6<br>C14H12O4 | 329.9958 | 1.3 | | 449.1633 | 7.64 | C15H25N6O10<br>C29H23NO4 |
| 131.0589 | 1.5 | C5H9NO3 | 249.99 | 1.85 | | 330.0526 | 1.55 | C17H14O5S | 463.2491 | 4.4 | |
| 132.0778 | 7.09 | C6H12O3 | 254.0857 | 1.56 | C9H18O6S | 331.0301 | 2.67 | | 463.2491 | 4 | |
| 136.0382 | 1.47 | C5H12S2;C5H4N4O<br>C4H8O5 | 257.9984 | 1.86 | | 331.1541 | 7.44 | C14H25N3O4S | 471.1453 | 7.64 | C20H21N7O7<br>C22H24F3NO7 |
| **147.0531** | **1.42** | **C5H9NO4** | 261.9917 | 3.52 | | 350.0937 | 1.42 | C16H18N2O5S | 473.1779 | 1.51 | C23H27N3O8<br>C20H31N3O8S |
| 148.0373 | 3.53 | C5H8O5<br>C6H12S2 | 264.0069 | 1.85 | | 369.0698 | 1.54 | C15H15NO10<br>C12H21NO8P2 | 484.2132 | 1.55 | C24H36O8S |
| 156.0039 | 1.84 | C6H4O5 | 266.024 | 1.41 | C8H10O10 | 373.0807 | 1.65 | | 490.1528 | 4.93 | C24H26O11 |
| 169.0367 | 1.43 | C7H7NO4 | 266.0415 | 4.9 | C12H10O7 | 374.0919 | 7.41 | C20H19ClO5<br>C16H15ClN6O3 | 504.0499 | 7.4 | C22H16O14<br>C19H20O14S |
| 172.0351 | 4.9 | C7H8O5 | 267.0126 | 1.43 | | 387.0426 | 1.91 | C18H13NO7S<br>C11H17NO12S | 518.0618 | 7.41 | |
| 174.0169 | 3.52 | C7H10OS2<br>C6H6O6 | 268.0208 | 1.86 | | 395.0628 | 1.65 | C11H18N5O7PS<br>C17H17NO8S | 524.0568 | 1.65 | C22H20O13S |
| 192.0275 | 1.34<br>3.52 | C6H8O7 | 271.9992 | 1.43 | C10H8O7S<br>C5H10N2O7P2 | 406.037 | 3.52 | C18H14O9S | 531.2365 | 4 | |
| 194.0323 | 3.52 | | 275.1131 | 1.65 | C10H17N3O6<br>C15H17NO4 | 408.0139 | 3.52 | C17H12O10S<br>C16H12N2O7S2 | 531.2367 | 4.4 | |
| 200.9789 | 1.43 | C3H7NO5S2 | 278.0937 | 5.43 | C10H18N2O5S;C18H14O3<br>C13H14N2O5 | 409.1318 | 7.44 | C14H23N3O11;C15H27N3O6S2<br>C19H23NO9 | 542.1171 | 7.41 | C30H22O10 |
| 207.0116 | 1.42 | | 286.1483 | 4 | | 415.1551 | 1.56 | | 546.0386 | 1.66 | |
| 213.8895 | 1.85 | | 296.0165 | 1.85 | | 417.2432 | 4.4 | | 548.3385 | 4 | |
| 214.0098 | 3.53 | | 297.0461 | 1.33 | C8H15N3O5S2 | 417.2432 | 4 | | 548.3386 | 4.4 | |
| 217.9748 | 1.85 | C4H10O4S3 | 297.0949 | 1.65 | C16H15N3OS | 422.0034 | 3.53 | | 574.1246 | 6.74 | C34H22O9<br>C27H26O14 |
| 218.0241 | 3.52 | C8H10O5S;C12H10S2<br>C11H6O5 | 299.974 | 1.85 | | 422.0744 | 7.4 | C12H22O14S<br>C20H19ClO8 | 612.155 | 4.9 | C20H32N6O12S2<br>C30H28O14 |
| 218.0797 | 6.61 | C9H14O6 | 303.1562 | 1.61 | C11H21N5O5<br>C16H21N3O3 | 426.0901 | 1.66 | C13H22N4O8S2<br>C22H18O9 | 614.032 | 3.55 | |
| 220.0174 | 4.9 | | 308.0156 | 3.56 | C10H16N2OS4<br>C10H12O9S | 429.995 | 3.53 | C22H22ClNO6 | | | |

**Supplementary Table 2.** Isotopologue assignation to exact mass.

| Metabolite | Theoretical mass | Experimental mass (neutral) | Mass shift (ppm) |
|---|---|---|---|
| $^{13}C_1$-Glu | 148.0565126 | 148.05674 | 2 |
| $^{15}N$-Glu | 148.0501927 | 148.05005 | 1 |
| $^{13}C_5$-Glu | 152.069932 | 152.06998 | -0.3 |
| $^{13}C_4$,$^{15}N$-Glu | 152.063612 | not found | |

**Supplementary Table 3.** Donor age and functional data of primary culture human hepatocytes used in this study. Values are expressed as pmol/min/million cells and quantified as described in Lahoz et al (Current Drug Metabolism, 2008, 9, 12-19) and Donato et al (Anal Bioanal Chem, 2010, 396, 2251-2263).

| | Donor data | | | Cell culture data | | | | | | | | |
|---|---|---|---|---|---|---|---|---|---|---|---|---|
| | **Age** | **Gender** | **Cause of death** | **Viability** | **CYP1A2** | **CYP2A6** | **CYP2B6** | **CYP2C9** | **CYP2D6** | **CYP2E1** | **CYP3A4** | **UGT1A1** |
| **PHH1** | 3 days | Female | Anoxia | 82% | n.d. | 855 | 153 | 4583 | 96 | 242 | 577 | 1.33 |
| **PHH2** | 15 years | Male | Anoxic encephalopathy | 60% | 0.3 | 254 | 25 | 3972 | 23 | n.d. | 184 | 14.6 |

n.d.: not determined

**Supplementary Table 4.** Age, gender and disease (diagnosis) of liver biopsy donors used for RNA control. Only healthy fragments were used for RNA isolation.

| | Age (yr) | Gender | Disease |
|---|---|---|---|
| **Donor 1** | 74 | Male | Hepatocarcinoma |
| **Donor 2** | 53 | Female | Liver metastasis |
| **Donor 3** | 54 | Male | Liver metastasis |
| **Donor 4** | 80 | Male | Cholangiocarcinoma |
| **Donor 5** | 61 | Male | Hepatocellular adenoma |

**Supplementary Table 5.** Antibodies used in this study.

| Antibody | Raised in | Dilution | Catalogue number |
|---|---|---|---|
| Hnf1A | Rabbit | 1/200 | Scbt-#8986 |
| Hnf1A | Goat | 1/200 | Scbt-#6547 |
| Hnf4 | Goat | 1/200 | Scbt-#6556 |
| Hnf4 | Rabbit | 1/200 | Scbt-#8987 |
| FoxA1/2 | Mouse | 1/200 | Scbt-#377033 |
| FoxA3 | Goat | 1/200 | Sc-5361 |
| hAlbumin | Goat | 1/500 | Bethyl A-80-229A |
| A1AT | Rabbit | 1/500 | Cell marque 223A-15 |
| AFP | Rabbit | 1/200 | Cell marque 203A-15 |
| Ki67 | Rabbit | 1/200 | Thermo R-9106 |
| Phalloidin-488 | | 1/50 | LifeTech A12379 |

**Supplementary Table 6.** qRT-PCR data used to generate heatmaps in Figure 2D and Figure 4E.

| | HDF-1 | HDF-2 | HDF-3 | HDF-4 | HDF-5 | HDF-6 | HDF-7 | HEP-L-1 | HEP-L-2 | HEP-L-3 | HEP-L-4 | HEP-L-5 | HEP-L-6 | HEP-L-7 | HUMAN LIVER |
|---|---|---|---|---|---|---|---|---|---|---|---|---|---|---|---|
| **GLS** | 13.83 | 4.72 | 6.59 | 6.39 | 6.19 | 5.45 | 6.57 | 7.92 | 10.13 | 4.72 | 5.12 | 4.24 | 3.72 | 3.86 | 1.00 |
| **GLS2** | 0.01 | 0.01 | 0.01 | 0.00 | 0.00 | 0.00 | 0.00 | 1.97 | 1.78 | 1.09 | 1.02 | 1.30 | 1.39 | 1.39 | 1.00 |
| **GLUL** | 9.55 | 3.15 | 3.72 | 3.76 | 2.83 | 3.51 | 2.94 | 1.72 | 1.78 | 0.89 | 1.16 | 1.04 | 1.08 | 1.39 | 1.00 |
| **GLUD1** | 0.11 | 0.08 | 0.09 | 0.06 | 0.05 | 0.06 | 0.06 | 0.28 | 0.28 | 0.14 | 0.14 | 0.16 | 0.16 | 0.27 | 1.00 |
| **GOT1** | 0.38 | 0.18 | 0.18 | 0.19 | 0.16 | 0.10 | 0.10 | 0.72 | 0.83 | 0.30 | 0.31 | 0.33 | 0.52 | 0.82 | 1.00 |
| **GOT2** | 3.96 | 1.62 | 2.17 | 1.87 | 1.64 | 1.61 | 1.45 | 5.01 | 6.08 | 2.27 | 2.67 | 2.58 | 2.00 | 2.45 | 1.00 |
| **GPT1** | 0.01 | 0.00 | 0.00 | 0.00 | 0.00 | 0.00 | 0.00 | 0.06 | 0.04 | 0.01 | 0.01 | 0.01 | 0.04 | 0.04 | 1.00 |
| **GPT2** | 2.69 | 1.54 | 2.07 | 1.65 | 1.77 | 1.13 | 1.20 | 5.50 | 2.76 | 2.44 | 3.16 | 1.93 | 2.70 | 2.82 | 1.00 |
| **BCAT1** | 43.71 | 38.32 | 35.63 | 30.80 | 28.84 | 33.47 | 38.85 | 20.82 | 14.77 | 17.88 | 21.71 | 19.84 | 9.51 | 9.35 | 1.00 |
| **BCAT2** | 5.24 | 4.47 | 4.68 | 4.50 | 3.96 | 5.22 | 4.94 | 6.94 | 5.98 | 4.94 | 3.78 | 3.88 | 3.13 | 3.24 | 1.00 |
| **SLC1A1** | 1.14 | 0.51 | 0.67 | 0.60 | 0.52 | 0.49 | 0.43 | 1.87 | 2.33 | 0.65 | 1.00 | 0.90 | 1.34 | 1.69 | 1.00 |
| **SLC1A2** | 0.01 | 0.00 | 0.00 | 0.00 | 0.00 | 0.01 | 0.01 | 0.12 | 0.12 | 0.15 | 0.20 | 0.12 | 0.18 | 0.20 | 1.00 |
| **SLC38A3** | 0.00 | 0.00 | 0.00 | 0.00 | 0.00 | 0.00 | 0.00 | 0.00 | 0.00 | 0.00 | 0.00 | 0.00 | 0.00 | 0.00 | 1.00 |
| **SLC38A2** | 0.13 | 0.14 | 0.35 | 0.53 | 0.54 | 0.61 | 0.66 | 0.05 | 0.03 | 0.45 | 0.76 | 0.85 | 1.24 | 1.15 | 1.00 |
| **SLC1A4** | 1.43 | 0.98 | 1.06 | 1.13 | 0.93 | 0.62 | 0.65 | 0.56 | 0.42 | 0.40 | 0.63 | 0.48 | 0.29 | 0.34 | 1.00 |
| **SLC1A5** | 101.48 | 73.77 | 83.87 | 87.12 | 78.52 | 68.83 | 70.03 | 112.60 | 82.42 | 81.29 | 91.14 | 74.54 | 54.76 | 59.10 | 1.00 |
| **SLC25A13** | 0.05 | 0.04 | 0.04 | 0.03 | 0.03 | 0.03 | 0.03 | 0.21 | 0.18 | 0.11 | 0.09 | 0.10 | 0.14 | 0.14 | 1.00 |
| **SLC25A12** | 10.23 | 6.39 | 8.31 | 8.54 | 7.59 | 9.45 | 9.22 | 6.63 | 3.80 | 7.16 | 5.96 | 5.28 | 3.36 | 3.51 | 1.00 |
| **SLC25A22** | 3.22 | 2.54 | 2.30 | 2.09 | 1.99 | 3.41 | 3.27 | 2.20 | 1.58 | 2.19 | 1.54 | 1.29 | 0.64 | 0.76 | 1.00 |
| **SLC25A18** | 0.00 | 0.00 | 0.00 | 0.00 | 0.00 | 0.00 | 0.00 | 0.05 | 0.04 | 0.03 | 0.03 | 0.03 | 0.07 | 0.07 | 1.00 |
| **PYCR1** | 3.99 | 3.08 | 2.58 | 2.47 | 2.17 | 2.71 | 2.69 | 1.47 | 1.13 | 1.39 | 1.16 | 0.90 | 0.23 | 0.17 | 1.00 |
| **PYCR2** | 9.51 | 7.52 | 6.28 | 6.34 | 5.66 | 4.59 | 4.58 | 11.71 | 11.39 | 5.06 | 6.34 | 6.00 | 5.03 | 4.69 | 1.00 |
| **PYCR3** | 1.47 | 1.30 | 1.10 | 1.00 | 0.93 | 0.90 | 1.12 | 1.67 | 1.47 | 0.90 | 0.89 | 0.91 | 0.92 | 0.78 | 1.00 |
| **ALDH18A1** | 4.26 | 3.80 | 3.23 | 2.93 | 2.69 | 3.22 | 3.15 | 3.77 | 3.01 | 3.01 | 2.39 | 2.28 | 2.82 | 2.88 | 1.00 |
| **ALDH4A1** | 0.02 | 0.01 | 0.01 | 0.01 | 0.01 | 0.03 | 0.03 | 1.82 | 1.64 | 0.69 | 0.46 | 0.44 | 1.06 | 0.98 | 1.00 |
| **PRODH** | 0.04 | 0.01 | 0.01 | 0.01 | 0.01 | 0.00 | 0.00 | 0.05 | 0.04 | 0.09 | 0.04 | 0.04 | 0.15 | 0.13 | 1.00 |
| **OAT** | 5.26 | 4.59 | 4.41 | 4.26 | 3.78 | 5.70 | 5.70 | 6.77 | 6.00 | 3.61 | 3.72 | 3.56 | 7.44 | 7.16 | 1.00 |

**Supplemental Figure 1. HEP-L cells activate the hepatic program and perform basic hepatic functions**. **(A)** Regression analysis between gene expression levels depicted throughout this study and the expression levels included in the NCBI Gene Expression Omnibus (accession number GSE42643) (Huang et al., 2014). Data is represented as mean ± s.d. from at least three experiments with 3-4 biological replicates each. A total of 32 genes are depicted. **(B)** Representative fluorescence images of HDF and HEP-L cells immunostained with antibodies against HNF4, FOXA1/2 and $\alpha$-fetoprotein. Nuclei were stained with DAPI. Bar equals 50 µm.

**Supplemental Figure 2. Cell number and volume after cell reprogramming to HEP-L cells**. HDF were reprogrammed to HEP-L cells as described in experimental procedures. At day 12, cells were trypsinized and cell number and diameter determined manually in a Neubauer chamber and confirmed with a Z2 coulter counter analyzer. Cell volume was determined using the formula: $V = \frac{4}{3}\pi r^3$. A representative analysis is depicted. Data shown is represented as mean ± s.d. from three experiments with 3 biological replicates.

**Supplemental Figure 3. $^{13}C$ NMR spectra of the cell supernatant described in Figure 2B.**

**Supplemental Figure 4. Mass Isotopomer Distribution (MID) of intracellular glutamate.** Cells were incubated with HMM media containing 2mM $^{13}C_5{}^{15}N_2$-Gln for 24 hours and intracellular levels of glutamate isotopologues determined by LC-MS. Data is represented as mean ± s.d. from a total of 15 replicates per group.

**Supplemental Figure 5. HDF and HEP-L cells do not proliferate at day 12.** Human dermal fibroblasts were seeded on collagen coated plates and reprogrammed by infection with equal amounts of lentiviral vectors encoding HNF4A, HNF1A and FOXA3 or the corresponding empty vectors (HDF) as described in experimental procedures. Representative fluorescence images of HDF (2 and 12 days) and HEP-L cells (12

days) immunostained with FOXA3 and proliferative marker Ki67 are shown. Nuclei were stained with DAPI. Bar equals 50 µm.

**Supplemental Figure 6. Intracellular and extracellular concentration of glutamate in HDF and HEP-L cells.** Cells were incubated with HMM media containing 2mM $^{13}C_5{}^{15}N_2$-Gln for 24 hours. Extracellular and intracellular concentration of glutamate isotopologues were determined by LC-MS. Absolute intracellular glutamate concentration was estimated by dividing the total amount of glutamate in the cell plate expressed as milimoles by total cellular volume determined in parallel plates (average cell volume x plate cell number). Data is represented as mean ± s.d. from a total of 15 replicates per group.
